# Glucosinolates from Seed-Press Cake of *Camelina sativa* (L.) Crantz Extend Yeast Chronological Lifespan by Modulating Carbon Metabolism and Respiration

**DOI:** 10.3390/antiox14010080

**Published:** 2025-01-11

**Authors:** Francesco Abbiati, Ivan Orlandi, Stefania Pagliari, Luca Campone, Marina Vai

**Affiliations:** 1 Dipartimento di Biotecnologie e Bioscienze, Università di Milano-Bicocca, Piazza della Scienza 2, 20126 Milano, Italy; f.abbiati1@campus.unimib.it (F.A.); ivan.orlandi@unimib.it (I.O.); stefania.pagliari@unimib.it (S.P.); luca.campone@unimib.it (L.C.); 2 SYSBIO Centre of Systems Biology, 20126 Milano, Italy

**Keywords:** Camelina, glucosinolates, chronological aging, *Saccharomyces cerevisiae*, carbon metabolism, respiration

## Abstract

Glucosinolates (GSLs) are nitrogen/sulfur-containing glycosides widely present in the order of Brassicales, particularly in the Brassicaceae family. Camelina (*Camelina sativa* (L.) Crantz) is an oilseed plant belonging to this family. Its seeds, in addition to a distinctive fatty acid composition, contain three aliphatic GSLs: glucoarabin, glucocamelinin, and homoglucocamelinin. Our study explored the impact of these GSLs purified from Camelina press cake, a by-product of Camelina oil production, on yeast chronological aging, which is the established model for simulating the aging of post-mitotic quiescent mammalian cells. Supplementing yeast cells with GSLs extends the chronological lifespan (CLS) in a dose-dependent manner. This enhancement relies on an improved mitochondrial respiration efficiency, resulting in a drastic decrease of superoxide anion levels and an increase in ATP production. Furthermore, GSL supplementation affects carbon metabolism. In particular, GSLs support the pro-longevity preservation of TCA cycle enzymatic activities and enhanced glycerol catabolism. These changes contribute positively to the phosphorylating respiration and to an increase in trehalose storage: both of which are longevity-promoting prerequisites.

## 1. Introduction

Glucosinolates (GSLs) are a complex group of nitrogen/sulfur-containing glycosides, produced as secondary metabolites by a large number of plants belonging to the order of Brassicales, which includes the Brassicaceae family. In this family, there are many edible plants, such as Brussels sprouts, cauliflower, garden cress, cabbage, broccoli, and radish, in which the GSL content is particularly abundant [1,2,3]. To date, more than 130 different GSL structures have been well-documented [4]. Structurally, all GSLs have a common core structure characterized by a β-thioglucose linked by a sulfur atom to a (Z)-N-hydroximinosulfate ester and an amino acid-derived, variable side chain (R group) [2,4]. GSLs may be classified in subgroups according to several criteria. The most frequently used is based on the biosynthetic precursor amino acid that categorizes GSLs into aliphatic, aromatic, and indolic GSLs [2,4]. GSLs can be hydrolyzed by endogenous plant myrosinases. These are thioglucosideglucohydrolases (EC 3.2.1.147) and remove glucose of the core structure. The resulting aglicone is unstable and rearranges producing isothiocyanates or other breakdown products, the nature of which depend upon different factors, including the nature of the R group [4,5]. GSLs and/or their breakdown products play important roles in plant protection against biotic and abiotic stresses [6,7,8], as well as, in agriculture for their biofumigant activity [9]. In addition, these compounds can provide beneficial effects on human health attributed, among others, to antioxidant and anti-inflammatory properties [10,11,12].

Camelina (*Camelina sativa* (L.) Crantz), also known as gold-of-pleasure, false flax, or linseed dodder, is an ancient oilseed plant within the Brassicaceae family [13]. Its crops display interesting agronomic features, such as a good growth under different environmental conditions, rapidly maturing short-season forms (spring and winter cultivars), and low requirements for water, fertilizers, and pesticides [13,14,15,16,17]. Camelina seeds contain three aliphatic GSLs: glucoarabin (9-methyl-sulfinyl-nonyl-glucosinolate, GSL9), glucocamelinin (10-methyl-sulfinyl-decyl-glucosinolate, GSL10), and homoglucocamelinin (11-methyl-sulfinyl-undecyl-glucosinolate, GSL11) [18]. Furthermore, Camelina seeds have a distinctive content in fatty acids (in particular unsaturated ω-3 and ω-6), which makes Camelina oil well-suited for many industrial and nutritional products ranging from biodiesel and lotions to dietary supplements [19,20]. Following oil extraction, Camelina press cake (PC) is obtained as a by-product and can be employed as cheap protein-rich feed for cattle and poultry [21,22]. This application makes the entire Camelina supply chain a promising example of an environmentally and economically sustainable bio-based process. In this context, Camelina GSL9, GSL10, and GSL11, contained in the PC, have a great potential due to their antioxidant properties that are worth studying, in order to increase the edible PC valorization. Thus, the objective of the present study was to analyze the effects of these GSLs, purified from Camelina PC, in the budding yeast *Saccharomyces cerevisiae*. This single-celled eukaryote has been highly instrumental as a model system for many purposes from basic to biomedical research. For example, *S.cerevisiae*-based studies have led to the identification/characterization of the nutrient-sensing TOR pathway, that regulates stress, growth, metabolism, and aging from yeast to humans [23,24]. In addition, yeast has been employed to identify natural compounds with anti-aging properties that proved to be functional (exemplified by spermidine) when tested in mammalian systems [25,26].

Here, we report that GSL9, GSL10, and GSL11 display anti-aging properties promoting chronological lifespan (CLS) extension. Such an extension relies on a more efficient phosphorylating respiration state that leads, on the one hand, to a lower superoxide anion (O_2_^•−^) content and, on the other, to ATP increase. In addition, a pro-longevity metabolism toward trehalose storage takes place.

## 2. Materials and Methods

### 2.1. Preparation and Purification of GSL Extract

Camelina PC was provided by FlaNat Research srl (Milan, Italy) and GSLs were extracted following a published method [27] with slight modifications. PC was homogenized into a fine powder using a grinder (TUBE-MILL 100, IKA, Staufenim Breisgau, Germany). Powdered sample was resuspended in 96% ethanol (ratio: 1 gr/5 mL) and subjected to sonication (2 cycles of 5 min each). Supernatants were filtered through a 0.45 µm PTFE filter. After extraction, GSLs were purified by Solid Phase Extraction (SPE). The SPE Mega Bond Elut NH_2_ cartridges were activated with methanol and equilibrated with 1% acetic acid in water. The extract was loaded into the NH_3_^+^ SPE and GSL fraction was eluted with a solution of methanol with 2% NH_4_OH. The purified extract was then evaporated, dissolved in water, and freeze-dried. The lyophilized fraction was dissolved in water at 10 mg/mL and filtered with 0.22 µm PES syringe filter before UPLC-DAD-HRMS analysis.

### 2.2. Yeast Strain, Growth Conditions, and CLS Determination

The yeast strain W303-1A (*MATa ade2-1 his3-11,15 leu2-3,112 trp1-1 ura3-1 can1-100*) was grown in batches at 30 °C in minimal medium (Difco Yeast Nitrogen Base without amino acids, 6.7 g/L) with 2% *w*/*v* glucose and the required supplements added in excess: adenine, histidine, and uracile at 200 mg/L and leucine at 500 mg/L [28]. CLS was determined by clonogenic assays [29]. Colony-forming units (CFUs) were counted starting with 72 h (Day 3, first age-point) after the diauxic shift (Day 0). The number of CFUs on Day 3 was considered the initial survival (100%). Cell number, extracellular glucose, and ethanol were measured at different time points during growth in order to characterize the growth profile (exponential phase, diauxic shift, post-diauxic phase, and stationary phase) of the culture [28]. Cell number was determined using a Coulter Counter-Particle Count and Size Analyser [30]. Duplication time was calculated as in [30]. Survival integral, namely the area under the CLS curves, was determined according to [31]. Treatments were performed at Day 0. GSL9, GSL10, and GSL11 (purchased from Extrasynthese, Genay, France) were added at the final concentrations of 270, 640, and 90 µM, respectively. Nicotinamide (NAM, Sigma-Aldrich, Darmstadt, Germany) was added at 5 mM final concentration [32].

### 2.3. Dosage of Metabolites and Enzymatic Activities

At designated time points, aliquots of the yeast cultures were centrifuged, and both pellets (washed twice) and supernatants were collected and frozen at −80 °C until used. Rapid sampling for intracellular metabolite measurements was performed as described [28]. The concentrations of glucose, ethanol, acetate, citrate, succinate, malate, fumarate, and glycerol were determined using enzymatic assays (K-HKGLU, K-ETOH, K-ACET, K-SUCC, K-CITR, K-LMALR, and K-GCROL kits from Megazyme, Bray, Ireland, and MAK060 from Sigma-Aldrich, Darmstadt, Germany). Extraction and determination of intracellular trehalose according to [33]. The K-HKGLU kit was used to quantify the released glucose.

Isocitrate lyase (Icl1) activity was assayed as previously reported [28]. Estimation of succinate dehydrogenase (SDH) activity was performed according to [34] by measuring at 540 nm the formation of formazan due to tetrazolium salt reduction. Glycerol-3-phosphate dehydrogenase (Gut2) activity was determined according to [35]: spheroplasts were prepared with Zymolyase 20T (MP Biomedicals, Solon, OH, USA).

ATP was extracted as described [36] and quantified using the ATP determination kit (Molecular Probes, Thermo Fisher Scientific, Waltham, MA, USA). Cell dry weight was measured as in [37]. Total protein concentration was assayed using the Pierce^TM^ BCA Protein Assay Kit (Thermo Fisher Scientific, Waltham, MA, USA).

### 2.4. Subcellular Fractionation

Mitochondria were prepared as in [38] with minor modifications. Briefly, at designated time points, about 10^9^ cells were harvested by centrifugation and spheroplasts were prepared in the presence of Zymolyase 20T (MP Biomedicals, Solon, OH, USA). Spheroplasts were resuspended in ice-cold homogenization buffer (0.6 M sorbitol, 10 mM Tris-HCl, pH 7.4, 1 mM EDTA, 0.2% (*w*/*v*) bovine serum albumin (Sigma-Aldrich, Darmstadt, Germany), containing 1 mM phenylmethylsulfonyl fluoride (Sigma-Aldrich, Darmstadt, Germany) and Complete EDTA-free cocktail of protease inhibitors (Roche Diagnostic, GmbH, Manheim, Germany). Spheroplasts were homogenized with 20 strokes using a Dounce homogenizer (Sigma-Aldrich, Darmstadt, Germany). Then, the homogenate was centrifuged at 1500 rcf for 5 min to remove cell debris and nuclei. The supernatant was clarified by centrifugation at 4000 rcf for 5 min and, finally, pellets of crude mitochondria were collected at 12,000 for 10 min. Pellets of crude mitochondria and the corresponding supernatants were used to measure the concentrations of mitochondrial and cytosolic fumarate, respectively. Subcellular fractions were checked by Western analysis using anti-3-phosphoglycerate kinase mAb (22C5 from Molecular Probes, Invitrogen, Thermo Fisher Scientific, Waltham, MA, USA) as a cytosolic marker and anti-Tom40 Ab (H-300 from Santa Cruz Biotechnology, Dallas, TX, USA) as a mitochondrial one. Secondary antibodies were purchased from Amersham (Cytiva, Amersham, UK). Detection of Western blots as described [39].

### 2.5. Respiration Assays and Fluorescence Microscopy

The basal oxygen consumption of intact cells was measured at 30 °C using a “Clark-type” oxygen electrode (Oxygraph System, Hansatech Instruments, Nortfolk, UK) as previously reported [40]. The addition of 37.5 mM triethyltin bromide (TET, Sigma-Aldrich, Darmstadt, Germany) and 10 µM of the uncoupler carbonyl cyanide 3-chlorophenylhydrazone (CCCP, Sigma-Aldrich, Darmstadt, Germany) to the oxygraph chamber accounted for the non-phosphorylating respiration and the maximal/uncoupled respiratory capacity, respectively [32]. The addition of 2 M antimycin A (Sigma-Aldrich, Darmstadt, Germany) accounted for non-mitochondrial oxygen consumption. Respiratory rates for the basal oxygen consumption (J_R_), the maximal/uncoupled oxygen consumption (J_MAX_), and the non-phosphorylating oxygen consumption (J_TET_) were determined from the slope of a plot of O_2_ concentration against time, divided by the cell number. The net respiration (netR) was obtained by subtracting J_TET_ from J_R_. Index of respiratory competence (IRC) was determined as previously described [41,42]. At different time points, identical samples of the yeast cultures were plated on Yeast Extract Peptone/2% glucose (YEPD) agar plates and on YEP/3% *v*/*v* glycerol (YEPG) plates. IRC was calculated as colonies on YEPG divided by colonies on YEPD times 100%.

Dihydroethidium (DHE, Sigma-Aldrich, Darmstadt, Germany) staining was performed to analyze superoxide anion (O_2_^•−^) [43]. The mitochondrial membrane potential was assessed by staining with 3,3′-dihexyloxacarbocyanine iodide (DiOC_6_, Molecular Probes, Invitrogen, Thermo Fisher Scientific, Waltham, MA, USA) [44]. Cells were counterstained with propidium iodide to discriminate between live and dead cells. A Nikon Eclipse E600 fluorescence microscope equipped with a Nikon Digital Sight DS Qi1 camera was used. Digital images were acquired and processed using Nikon NIS-Elements BR 4.30.00 64-bit software, https://www.microscope.healthcare.nikon.com/products/software/nis-elements (accessed on 8 January 2025).

### 2.6. Statistical Analysis

All values are presented as the mean of three independent experiments ± standard deviation (SD). Three technical replicates were analyzed in each independent experiment. Statistical significance was assessed by one-way ANOVA test. The level of statistical significance was set at a *p* value of ≤0.05. All data were processed with Microsoft Excel 2019 (Microsoft Corporation, Redmond, WA, USA) to calculate the average values and standard deviations. Statistical analyses were performed using GraphPad Prism software version 9.5.1 (GraphPad Inc., La Jolla, CA, USA).

## 3. Results and Discussion

### 3.1. Characterization of GSL Extract

**Figure 1:**
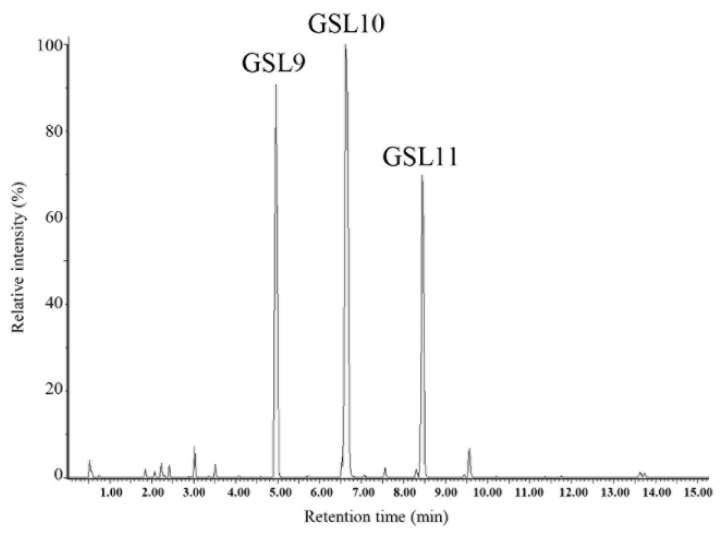
Representative UPLC-DAD- HRMS chromatogram of GSLs purified from Camelina press cake.

As a first step, we analyzed, by UPLC-DAD-HRMS, the content of the extract purified from Camelina PC (see Section 2), which was then used in the experiments with *S.cerevisiae* cells. A representative chromatogram is presented in Figure 1, showing that in the purified extract only three aliphatic GSLs were present, namely GSL9, GSL10, and GSL11.

### 3.2. GSL Extract Increases CLS

**Figure 2:**
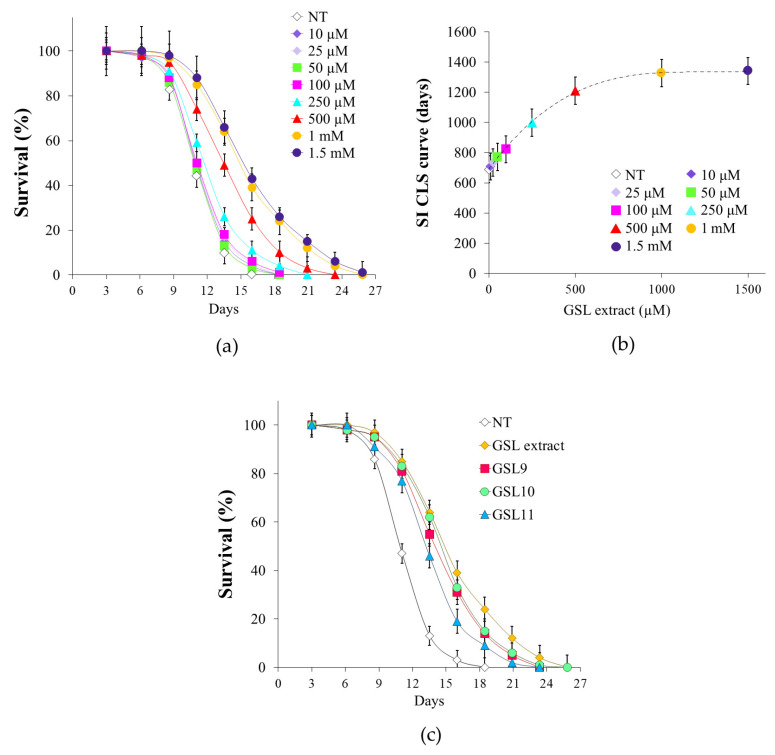
GSL extract supplementation at the diauxic shift promotes CLS longevity. Yeast cells were grown in minimal medium/2% glucose and the required supplements in excess (see Section 2). At the diauxic shift (Day 0), different concentrations (from 10 μM to 1.5 mM) of GSL extract were added and (**a**) survival over time of treated and untreated (NT) cultures was determined by colony-forming capacity on YEPD plates. 72 h after the diauxic shift (Day 3) was considered the first age point, corresponding to 100% survival. (**b**) Dose–response relationship of the survival integral values (SI) versus the concentrations of GSL extract. (**c**) CLS of cells supplied with GSL extract (1 mM) or GSL9, GSL10, and GSL11 separately, at the concentration which was present in 1 mM GSL extract. All data refer to mean values determined in three independent experiments with three technical replicates each. Standard deviations (SD) are indicated.

Taking advantage of the chronological aging model, which allows us to simulate in *S.cerevisiae* cellular aging of post-mitotic quiescent mammalian cells, we investigated whether the purified GSL extract may have any effect on yeast longevity as well as on cellular metabolism. To this end, in the context of a standard CLS experiment [45], GSL extract was supplemented to cells in a range of different concentrations (from 10 µM to 1.5 mM). Supplementation was done at the diauxic shift (Day 0) because it is at this point that a massive metabolic reconfiguration takes place enabling cells to acquire a set of features required for survival during the quiescent state [46,47]. GSL extract extended CLS (Figure 2a) showing a dose–response relationship between the increase of both mean and maximum CLS (Table 1) as well as of the survival integral (Table 1 and Figure 2b) and the concentrations of GSL extract. As shown in Table 1 and in the dose–response curve of Figure 2b, the maximal benefit for CLS extension was achieved for 1 mM GSL extract, whilst higher concentration such as 1.5 mM could not further enhance cell longevity. Consequently, in this study, 1 mM GSL extract was chosen to investigate the pro-longevity effect of GSL extract on chronological aging. Furthermore, since the GSL extract obtained from Camelina PC only consisted of three GSLs, namely GSL9, GSL10, and GSL11 (Figure 1), we also evaluated the effects on CLS of the three GSLs separately using the amount of each that was present in 1 mM GSL extract. All three GSLs supplied separately increased CLS (Figure 2c), confirming the pro-longevity effect exerted by the GSL extract.

### 3.3. GSL Extract Preserves Mitochondrial Functionality

**Figure 3:**
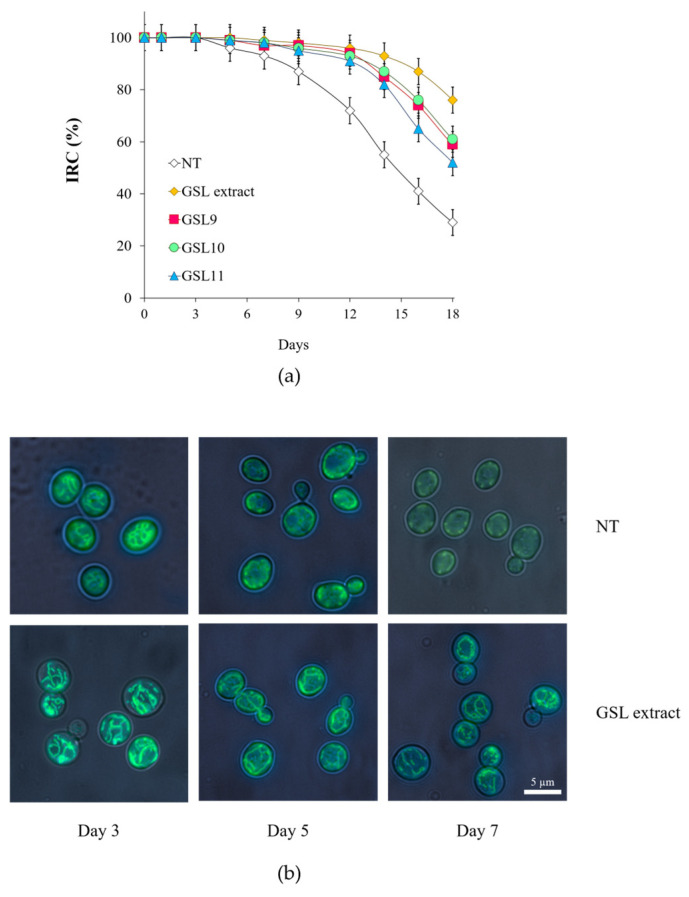
GSL extract supplementation at the diauxic shift preserves mitochondrial functionality. Cells were grown and supplied with GSL extract (1 mM) as in Figure 2 and (**a**) starting from Day 0, aliquots of the indicated cultures were serially diluted and plated onto YEPD and YEPG plates in order to determine the index of respiratory competence (IRC). All data refer to mean values determined in three independent experiments with three technical replicates each. Standard deviations (SD) are indicated. (**b**) Representative images of NT and GSL extract-supplemented cultures stained with DiOC_6_ to visualize mitochondrial membranes at the indicated time points.

Longevity is tightly linked to mitochondrial functionality. Indeed, increase of mitochondrial dysfunction is one feature that has been observed in aging across species and decline of mitochondrial functionality is considered a hallmark of aging [48,49,50]. In *S.cerevisiae*, mitochondrial functionality can be assessed by measuring the IRC, which defines the percentage of viable cells competent to respire [41]. At the diauxic shift, both the unsupplemented culture and that supplemented with the GSL extract were respiration-competent, showing an IRC of about 100% (Figure 3a). Afterwards, as expected [32,51], a time-dependent loss of mitochondrial functionality was observed with increasing chronological age. However, at Day 18 the IRC of the supplemented culture was still about 75% against about 30% of the unsupplemented one (Figure 3a). A similar behavior was observed when GSL9, GSL10, and GSL11 were supplied separately (Figure 3a), indicating that GSLs preserve mitochondrial functionality. In addition, as cells age, mitochondria undergo a gradual loss of the membrane potential along with morphological changes: the mitochondrial tubular network becomes punctiform (also referred to as mitochondrial fragmentation) [52,53]. Fluorescent staining with DiOC_6_ dye, which accumulates specifically at mitochondrial membranes depending on their membrane potential [44], revealed that already at Day 5 the mitochondrial network of chronologically aging cells underwent fragmentation and punctuated structures appeared (Figure 3b). On the contrary, following GSL extract supplementation, tubular shapes with bright fluorescence were still present at Day 7 (Figure 3b), suggesting that the mitochondrial functionality is preserved in line with IRC results.

### 3.4. GSL Extract Supplementation at the Diauxic Shift Correlates with a More Efficient Respiration

**Figure 4:**
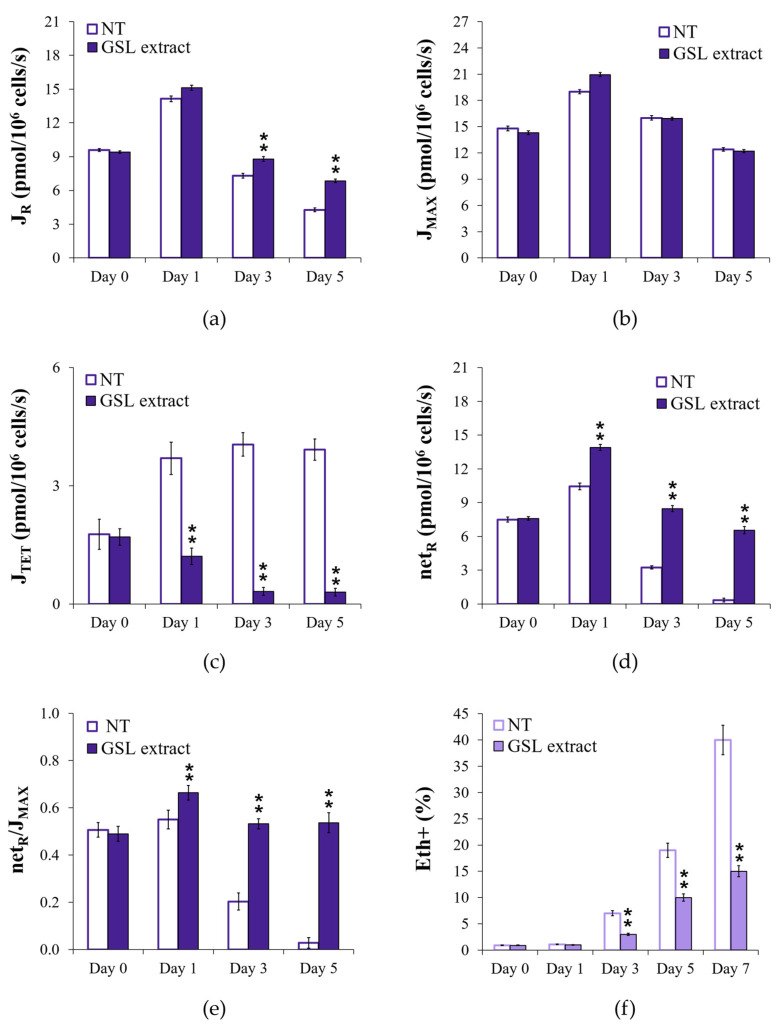
GSL extract supplementation at the diauxic shift promotes phosphorylating respiration. Cells were grown and supplied with GSL extract (1 mM), as in Figure 2, and oxygen uptake rates (J) are expressed as pmol/10^6^ cells/s. (**a**) Basal respiration rate (J_R_), (**b**) uncoupled respiration rate (J_MAX_), (**c**) non-phosphorylating respiration rate (J_TET_), (**d**) net respiration (netR = J_R_ − J_TET_), and (**e**) fraction of the electron transfer system utilized for ATP synthesis (netR/J_MAX_) were measured at the indicated time points. Substrates and inhibitors used in the measurements of the respiratory parameters are detailed in the text. (**f**) Bar charts of the percentage of fluorescent/superoxide positive cells assessed by the superoxide-driven conversion of non-fluorescent dihydroethidium into fluorescent ethidium (Eth). Day 0, diauxic shift. All data refer to mean values determined in three independent experiments with three technical replicates each. SD is indicated. Statistical significance as assessed by one-way ANOVA test is indicated (** *p* ≤ 0.01).

Considering that following the diauxic shift a respiration-based metabolism takes place and given the influence played by respiration on CLS [54,55,56], we quantified some respiratory parameters in both supplemented and unsupplemented cultures. When cells were supplied with the GSL extract at the diauxic shift, a slight increase in basal oxygen consumption (J_R_) was observed, whilst maximal respiratory capacity (J_MAX_) was unaffected (Figure 4a,b). Since J_MAX_ was assayed in the presence of the protonophore CCCP, which dissipates the proton gradient across the mitochondrial membrane, it follows that the membrane potential is not influenced by GSL supplementation. Carbon starvation elicits a transition from phosphorylating to non-phosphorylating respiration and an increase in oxidative damage [57]. Interestingly, in supplemented cells, the non-phosphorylating respiration (J_TET_) was extremely lower than that of the unsupplemented ones (Figure 4c). In such a determination, TET was used to inhibit ATP synthase, allowing oxygen measurement in a condition where the dissipation of the proton gradient due to ATP synthase-driven proton translocation is inhibited and only proton leak takes place. Consequently, the net respiration (netR = J_R_ − J_TET_), which assesses the coupled respiration, in supplemented cells was higher than that of the unsupplemented ones, especially 5 days after the diauxic shift where netR for the unsupplemented culture was reduced to values close to zero (Figure 4d), indicating that GSL supplementation seems to promote a more efficient coupling of electron transport to ATP generation. In addition, in supplemented cells the value of the ratio between netR and J_MAX_, which estimates the fraction of the electron transfer system utilized to drive ATP synthesis [58], was also significantly higher. This was particularly evident as a function of time in culture (Figure 4e), showing that GSL supplementation allows chronologically aging cells to retain a mitochondrial respiration toward a more coupled state for a longer period.

It is well known that electrons may accumulate at intermediate levels of the electron transport chain (ETC) favoring electron leakage. This, in turn, impacts on ROS formation as oxygen can readily accept single electrons generating O_2_ reduction intermediates, among which superoxide anions (O_2_^•−^). In excess, this radical, directly or converted to other ROS, causes frequently irreversible damage to cellular macromolecules, such as lipids, proteins, and DNA, contributing to the aging process [49,59,60]. GSL supplementation drastically reduced the expected increase of O_2_^•−^ that occurs as cells chronologically age (Figure 4f), in line with the low J_TET_ values measured in the supplemented cells since non-phosphorylating respiration is prone to generate O_2_^•−^ [57]. In addition, a lower level of O_2_^•−^ produced in the ETC decreases the risk of impairing mitochondrial functionality. This is what takes place in the supplemented cells, the mitochondria of which are functional and with a tubular morphology (Figure 3b). All this correlates with an enhanced CLS.

### 3.5. GSL Extract Supplementation at the Diauxic Shift Preserves TCA Enzymatic Activities

**Figure 5:**
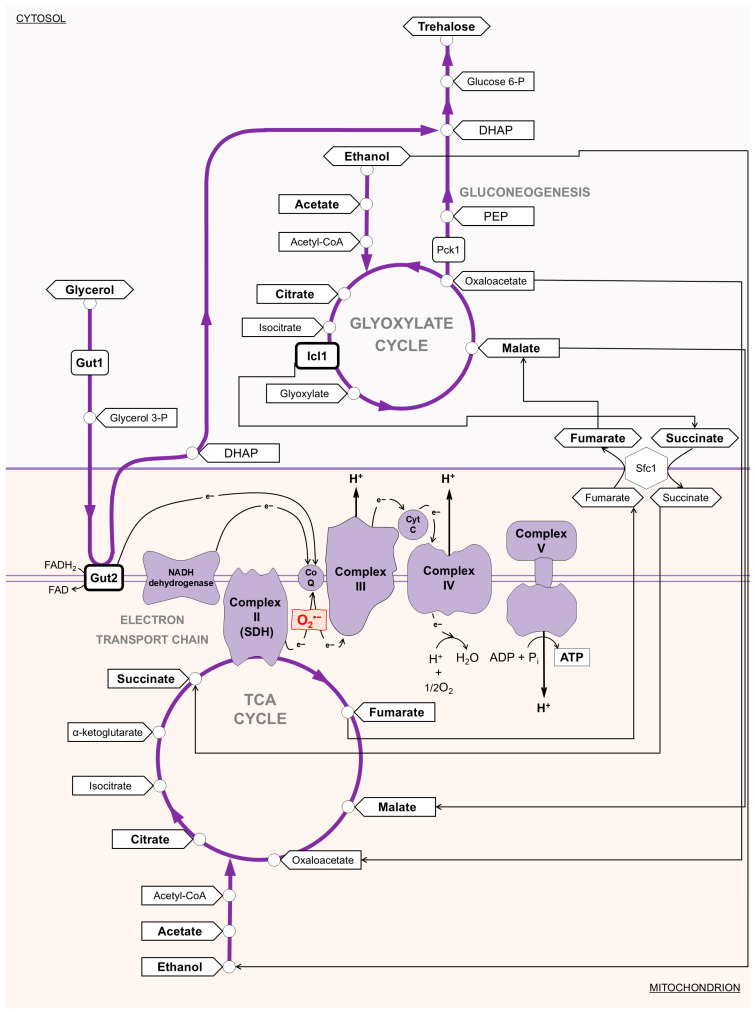
Scheme of metabolic pathways involved in utilizing the main non-fermentable carbon sources during chronological aging. The electron transport chain and three pathways (the glyoxylate shunt, TCA cycle, and gluconeogenesis) are schematically shown. Icl1, isocitrate lyase; Pck1, phosphoenolpyruvate carboxykinase; Gut1, glycerol kinase; Gut2, mitochondrial glycerol-3-phosphate dehydrogenase; Sfc1, mitochondrial succinate-fumarate transporter; SDH, succinate dehydrogenase complex; PEP, phosphoenolpyruvate; DHAP, dihydroxyacetone phosphate.

**Figure 6:**
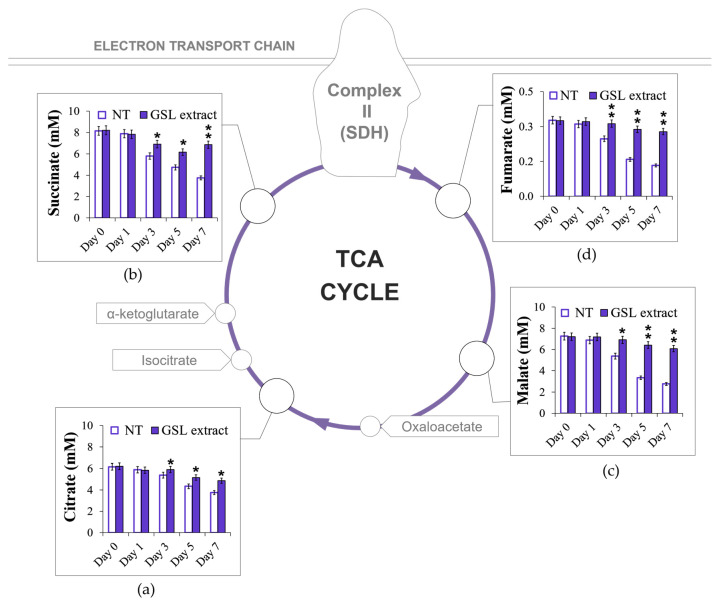
GSL supplementation at the diauxic shift preserves TCA intermediate contents. Cells were grown and supplied with GSL extract (1 mM), as in Figure 2, and intracellular concentrations of (**a**) citrate, (**b**) succinate, (**c**) malate, and (**d**) fumarate were measured at the indicated time points. Day 0, diauxic shift. SDH, succinate dehydrogenase complex. All data refer to mean values determined in three independent experiments with three technical replicates each. SD is indicated (* *p* ≤ 0.05 and ** *p* ≤ 0.01).

Mitochondria host the TCA cycle that fulfils a broad range of metabolic activities, from the oxidative generation of reducing equivalents that drive aerobic respiration, to providing building blocks for macromolecule synthesis. TCA enzymatic activities change at the diauxic shift when cellular metabolism shifts from fermentation to respiration, as well as during chronological aging [49,61,62]. Thus, initially, we measured the levels of some intermediates of the TCA cycle, namely citrate, succinate, malate, and fumarate that also have a metabolic connection with the glyoxylate shunt (Figure 5). The latter is activated after glucose depletion and is responsible for the generation of C4 units from C2 ones (ethanol and acetate) by excluding the two decarboxylation steps of the TCA cycle and also operates as an anaplerotic device of the TCA cycle [61,63]. After the diauxic shift, in the unsupplemented culture, the levels of citrate, succinate, malate, and fumarate decreased (Figure 6a–d) as expected [28,42]. On the contrary, in the GSL-supplemented culture, the levels of these intermediates remained higher as cells aged (Figure 6a–d). With regard to succinate, it is released from the glyoxylate shunt into the cytoplasm as a net product and is imported into the mitochondria to feed the TCA cycle [64].

**Figure 7:**
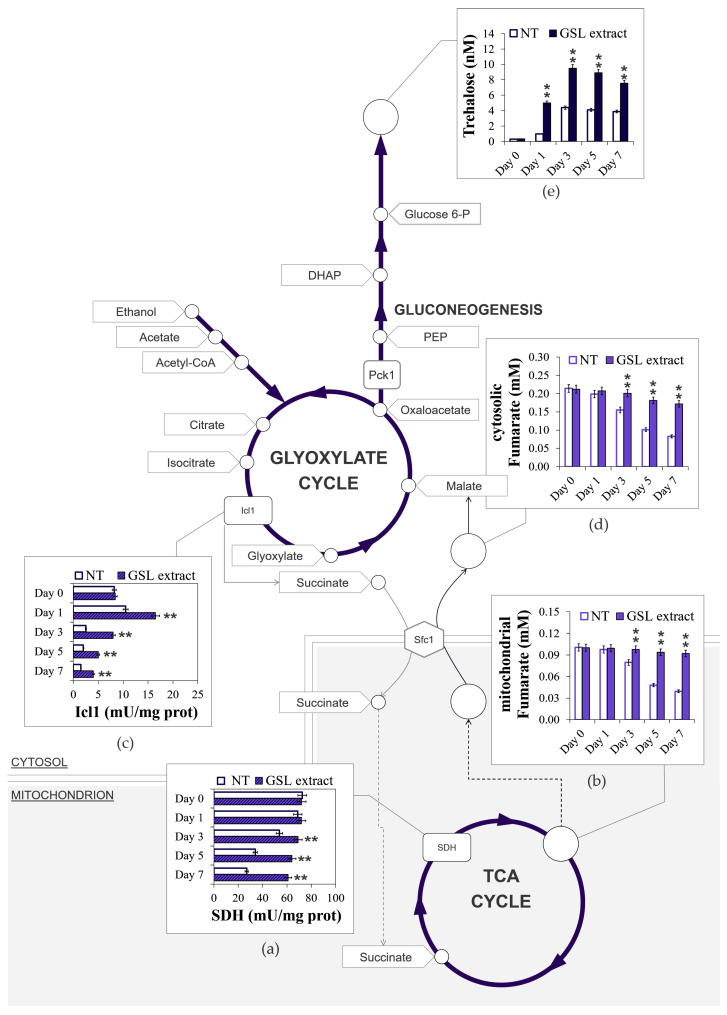
GSL extract supplementation at the diauxic shift promotes trehalose storage. At the indicated time points, (**a**) SDH enzymatic activity, (**b**) mitochondrial fumarate, (**c**) Icl1 enzymatic activity, (**d**) cytosolic fumarate, and (**e**) trehalose content evaluated for both treated and untreated cultures of Figure 2. Day 0, diauxic shift. Icl1, isocitrate lyase; Pck1, phosphoenolpyruvate carboxykinase; Sfc1, mitochondrial succinate-fumarate transporter; SDH, succinate dehydrogenase complex; PEP, phosphoenolpyruvate; DHAP, dihydroxyacetone phosphate. All data refer to mean values determined in three independent experiments with three technical replicates each. SD is indicated (** *p* ≤ 0.01).

In the latter succinate is oxidized to fumarate by succinate dehydrogenase complex (SDH), also known as Complex II (Figure 5 and Figure 6). This reaction is coupled to the reduction of ubiquinone to ubiquinol, which is the substrate for Complex III in the ETC; in such a way, SDH functionally links the activity of the TCA cycle to the ETC. Starting from the diauxic shift, SDH activity significantly decreased in unsupplemented cells (Figure 7a), in concert with a decrease in the levels of mitochondrial fumarate (Figure 7b) indicative of an aging-associated decline of the TCA cycle flux. In the GSL-supplemented culture SDH enzymatic activity and mitochondrial fumarate content were stably maintained at higher levels (Figure 7a,b). This indicates that GSL extract preserves TCA functioning, which is a feature that is involved in conferring longevity in yeast and other organisms [65,66,67,68]. In addition, in the context of chronological aging, mutants deleted in genes encoding subunits of SDH, namely *SDH1*, *SDH2*, and *SDH4*, show the shortest-lived phenotype among 33 single ETC component-deleted strains and higher O_2_^•−^ content associated with an impaired mitochondrial efficiency [69]. Interestingly, SDH has a medical significance, considering that its activity has been reported to decline with age in many tissues (brain, liver, heart, and skin) and to be reduced in some age-related diseases, including neurodegenerative disorders. This decline/reduction is associated with an increase of ROS contributing to cellular damage [70,71,72].

### 3.6. GSL Extract Supplementation at the Diauxic Shift Enhances Glyoxylate/Gluconeogenic Flux and Increased Trehalose Stores Without Affecting Ethanol/Acetate Catabolism

The glyoxylate shunt is composed of five enzymatic reactions, three of which are also present in the TCA cycle, whilst two are unique to the shunt. One of these is that catalyzed by isocitrate lyase (Icl1). This enzyme is solely localized in the cytosol and generates succinate and the name-giving metabolite glyoxylate from isocitrate (Figure 5 and Figure 7). Measurements of Icl1 enzymatic activity revealed a clear increase in the culture supplemented with GSLs compared with the unsupplemented counterpart (Figure 7c) indicative of an enhancement of the shunt. As stated above, cytosolic succinate is imported into the mitochondria. However, its transfer by the Sfc1 transporter provides cytosolic fumarate. In fact, fumarate is generated exclusively in the TCA cycle. Once in the cytosol fumarate is converted to malate and utilized to refill the glyoxylate shunt and fuel gluconeogenesis (Figure 5 and Figure 7) [73]. The latter, in turn, fuels the synthesis of storage carbohydrates, the accumulation of which contributes to the longevity of chronologically aging cells [54,74]. Following GSL supplementation, cytosolic fumarate levels increased, as well as trehalose content (Figure 7d,e). It is well recognized that the ability of chronologically aging cells to accumulate sufficient trehalose stores ensures, on the one hand, long-term survival during the stationary phase and, on the other, the resumption of growth upon nutrient supply. Hence, an enhancement of intracellular trehalose stores correlates with CLS extension [32,39,74,75]: this is also the case of the culture supplemented with the GSL extract (Figure 2a and Figure 7e).

**Figure 8:**
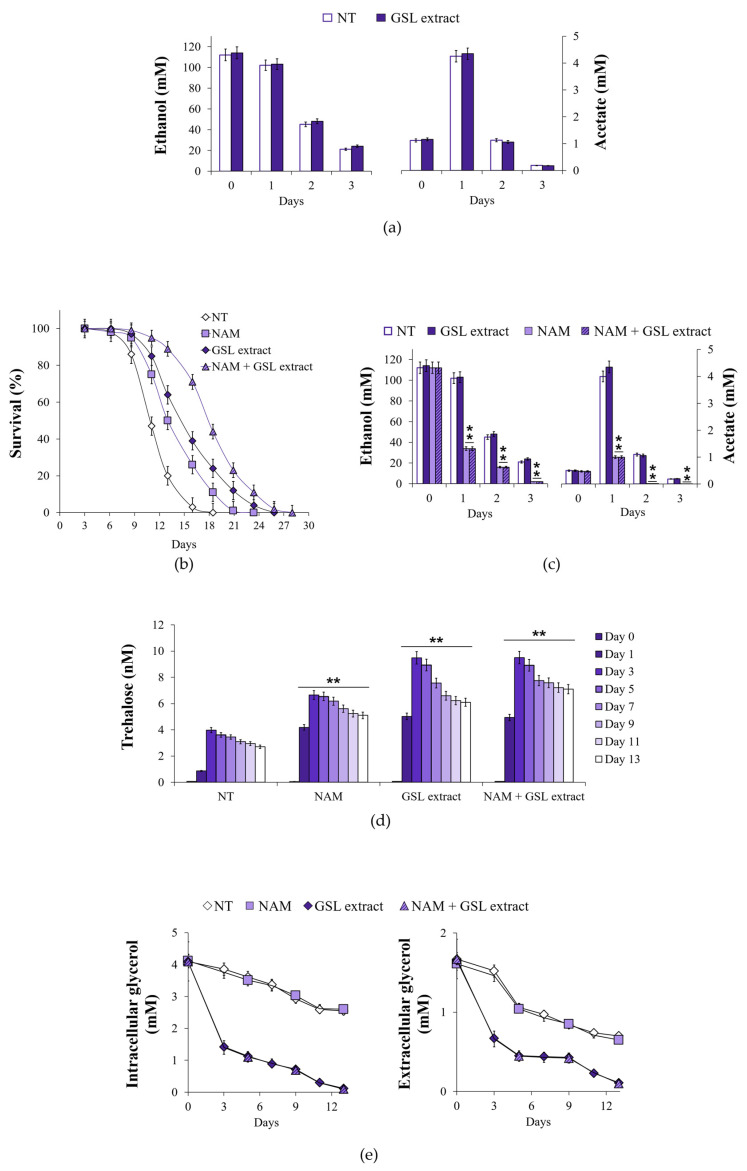
GSL extract supplementation at the diauxic shift promotes glycerol catabolism resulting in increased accumulation of trehalose and CLS extension. At the indicated time points, (**a**) bar charts of ethanol and acetate levels evaluated for both GSL extract-treated and untreated cultures of Figure 2. (**b**) CLS of cultures supplemented at Day 0 with NAM (5 mM), GSL extract (1 mM), or NAM + GSL extract. In parallel, (**c**) extracellular ethanol and acetate content, (**d**) intracellular trehalose concentration, and (**e**) intracellular and extracellular glycerol levels were measured. Day 0, diauxic shift. All data refer to mean values determined in three independent experiments with three technical replicates each. SD is indicated (** *p* ≤ 0.01).

During the diauxic shift, the glyoxylate shunt is fed by two C2 by-products of the fermentation, namely ethanol and acetate (Figure 5). No difference was observed in the utilization of extracellular ethanol and acetate following GSL supplementation (Figure 8a), suggesting that GSLs did not affect the catabolism of these compounds. Since, our published data indicated that supplementation at the diauxic shift of nicotinamide (NAM), a form of vitamin B3, extends CLS in concert with an enhancement of the glyoxylate shunt and increased ethanol/acetate catabolism (Figure 8b,c) [32], we analyzed the effects of a combined supplementation of GSL extract and NAM. An additive extension on CLS was produced when GSLs and NAM were provided together at the diauxic shift compared to single supplementations (Figure 8b and Table 2). On the other hand, the fast kinetics of ethanol/acetate depletion in the medium of NAM-supplemented cells was unaffected by GSL supplementation (Figure 8c). A feature of NAM-supplemented cells was also an increase in trehalose content compared with the unsupplemented ones [32], albeit to a lesser degree than that measured for GSL supplementation (Figure 8d). Supplementing NAM and GSLs together resulted in higher trehalose levels than those with GSL extract alone as cells age (Day 9 to Day 13) (Figure 8d). NAM is a non-competitive inhibitor of Sir2 activity [76] and, in the context of chronological aging, its supplementation inhibits Sir2-mediated deacetylation of phosphoenolpyruvate carboxykinase (Pck1) [32]. Pck1 catalyzes the main flux-controlling step of the gluconeogenesis, is active in the acetylated form [77] and is required for the utilization of ethanol/acetate. *SIR2* deletion or inhibition of its enzymatic activity lead to an improved assimilation of these C2 units by the glyoxylate-requiring gluconeogenesis resulting in increased accumulation of trehalose and longevity extension [32,78,79]. Given the results described above with single/combined supplementations of NAM and GSLs, we may reasonably rule out that Sir2 is the target of GSLs and that other targets/pathways can account for their effects.

### 3.7. Glycerol Catabolism Is Enhanced in GSL-Supplemented Chronologically Aging Cells in Concert with ATP Increase

**Figure 9:**
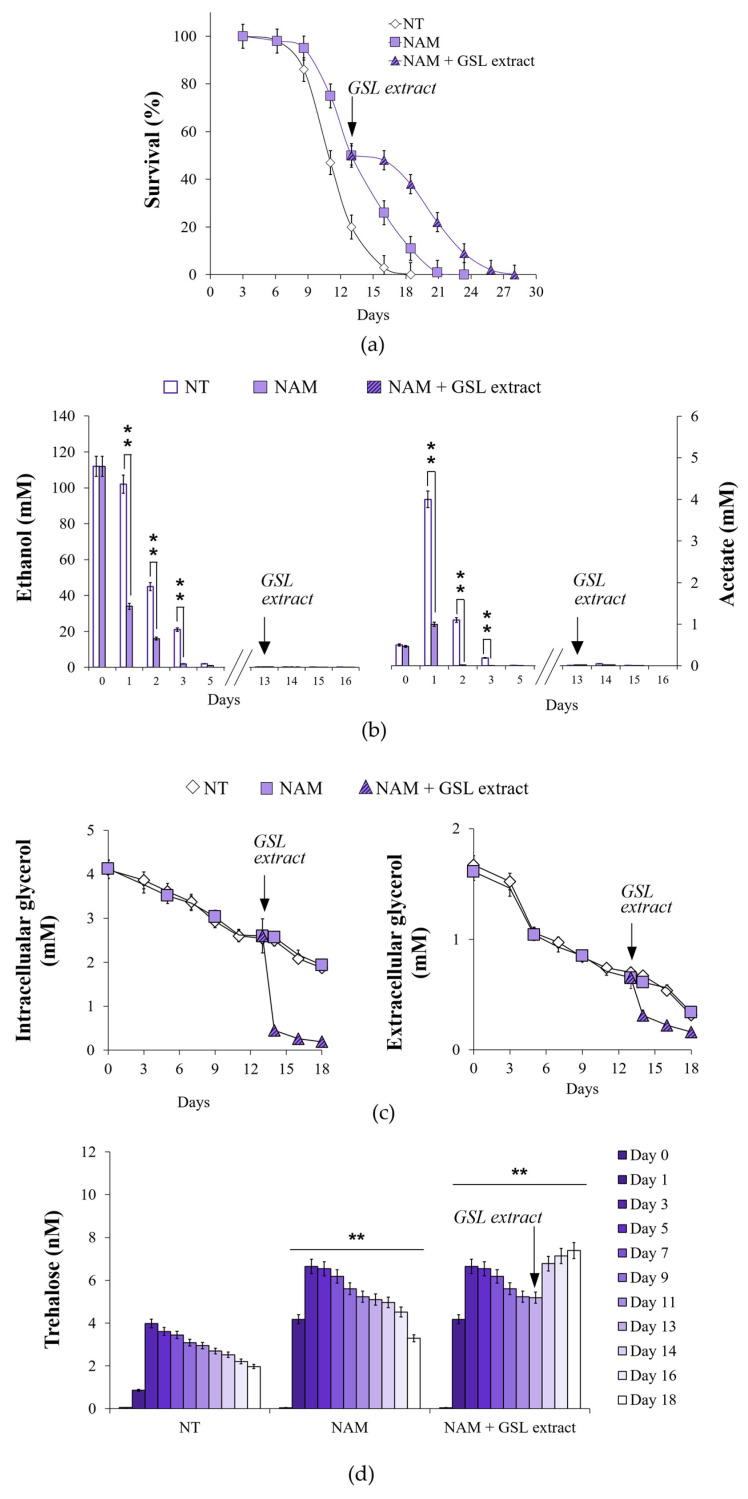
GSL extract supplementation during chronological aging further extends the CLS of NAM-treated cells. Wt cells were grown as in Figure 2 and supplied with NAM (5 mM) at Day 0. At the time point where NAM stationary cultures showed 50% of survival (mean CLS), GSL extract (1 mM) was added. (**a**) CLS of the indicated cultures. In parallel, (**b**) extracellular ethanol and acetate content, (**c**) intracellular and extracellular glycerol levels, and (**d**) intracellular trehalose concentration were measured. All data refer to mean values determined in three independent experiments with three technical replicates each. SD is indicated (** *p* ≤ 0.01).

The L-glycerol 3-phosphate (L-G3P) pathway becomes operative at the diauxic shift, allowing glycerol utilization [80]. Glycerol is a C3 by-product of yeast fermentation and, by the L-G3P pathway, is catabolized to dihydroxyacetone phosphate that can be used to fuel gluconeogenesis downstream of the step catalyzed by Pck1 (Figure 5). In GSL-supplemented chronologically aging cells, intracellular and extracellular glycerol decreased more rapidly than in unsupplemented cells, as well as in NAM-supplemented ones (Figure 8e). The kinetics of glycerol utilization following the combined supplementation of NAM and GSLs was similar to that of GSLs alone (Figure 8e). To further examine the effects on glycerol catabolism of GSLs, the latter were added to NAM-supplemented chronologically aging cells when the culture reached 50% of survival (mean CLS) (Figure 9a). At this time point, extracellular ethanol and acetate were completely depleted (Figure 9b), whilst glycerol was still present (Figure 9c). GSL supplementation determined a strong decrease in intracellular and extracellular glycerol (Figure 9c). This decrease corresponded temporally to the increase in trehalose, the content of which started to rise again reaching levels higher than those of the single NAM supplementation (Figure 9d). All these changes in the carbon metabolism were accompanied by the extension of CLS (Figure 9a). Taken together all these data indicate that the GSL extract, on the one hand, specifically enhances glycerol catabolism and, on the other, that glycerol utilization is directed toward trehalose biosynthesis.

**Figure 10:**
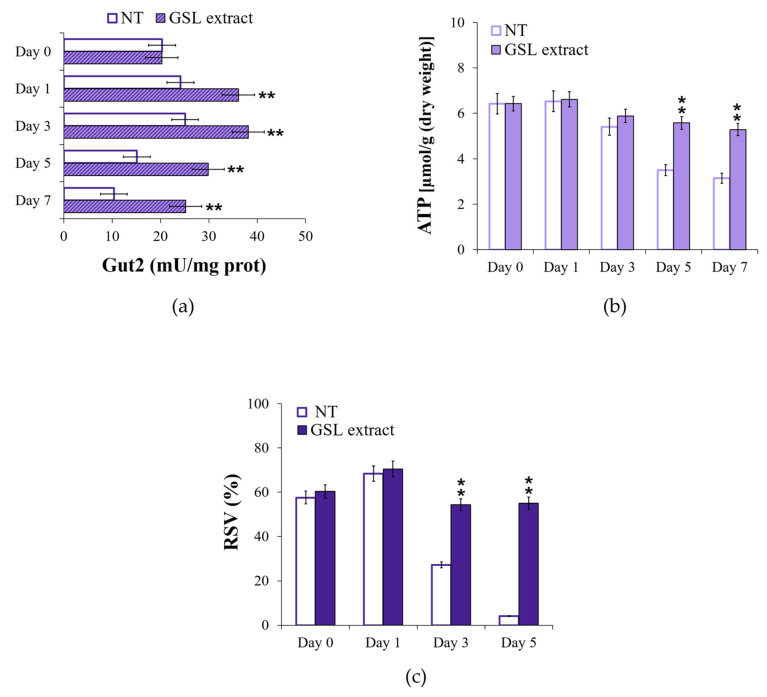
GSL extract supplementation at the diauxic shift positively affects ATP levels. At the indicated time points, (**a**) Gut2 enzymatic activity and (**b**) ATP content were evaluated for both treated and untreated cultures of Figure 2. For the same cultures: (**c**) bar charts of the respiration state value (RSV = [netR / (J_MAX_ − J_TET_)] × 100). Day 0, diauxic shift. All data refer to mean values determined in three independent experiments with three technical replicates each. SD is indicated (** *p* ≤ 0.01).

The enhancement of glycerol catabolism following GSL supplementation was further assessed by measuring the enzymatic activity of the FAD-dependent glycerol-3-phosphate dehydrogenase Gut2. In effect, glycerol catabolism specifically requires Gut1, a cytosolic glycerol kinase that phosphorylates glycerol to glycerol-3-phosphate (G3P). The latter is oxidized to DHAP with a concurrent reduction of FAD to FADH_2_ by Gut2, which is located in the mitochondrial membrane (Figure 5) [80]. In GSL-supplemented chronologically aging cells, Gut2 activity was higher than that in unsupplemented cells (Figure 10a), matching the increase of glycerol depletion (Figure 8e). It is known that the electron pair of FADH_2_ is transferred to the mobile electron carrier coenzyme Q, and via the latter to Complex III of the ETC. Electron flux, the final step of which is catalyzed by Complex IV, is coupled to proton pumping across the inner membrane of the mitochondria. The resulting proton motive force fuels ATP synthesis (Figure 5). Measurements of ATP levels showed that, in GSL-supplemented chronologically aging cells, these levels remained higher than those in unsupplemented ones (Figure 10b). These results mirror respiratory results (Figure 4c,d) which pointed to a more efficient coupling of electron transport to ATP generation following GSL supplementation.

In keeping with this, the respiration state value (RSV), which represents the percentage of stimulation of oxidative phosphorylation compared to basal respiration capacity [57], was higher in GSL-supplemented cells than that in unsupplemented ones (Figure 10c) which is indicative of an increase in the oxidative phosphorylation efficiency. In this context, we can hypothesize, although yet fully speculative, that the increase in Gut2 activity together with that of SDH (Figure 7a), both by delivering electrons to the ETC via ubiquinone, might increase the electron flux through the ETC. This might reduce electron stalling in the ETC decreasing the probability of unpaired electron leakage to produce ROS. In particular, Complex III generates O_2_^•−^ during the Q-cycle linked to the formation of an unstable semiquinone intermediate, which can donate its unpaired electron to oxygen. In general, under some circumstances, increasing the electron flux through the ETC decreases ROS formation [81]. Furthermore, a more efficient electron transfer to ubiquinone by channeling the electrons via the tightly bound FADH_2_ directly to the respiratory chain might explain the increase in its efficiency. In this regard, it has been observed that the yeast respiratory chain efficiency increased upon raising the growth temperature (from 30 °C up to 37 °C) and this temperature-dependent increase required Gut2 [82].

## 4. Conclusions

The GSL extract, which has been purified by us from a seed-press cake of *C. sativa*, displays a pro-longevity effect for yeast cells experiencing CLS. The increase of both mean and maximum CLS is also observed when the three aliphatic GSLs (GSL9, GSL10, and GSL11), which make up the extract, were supplied separately. CLS extension occurs since GSLs are effective in preserving mitochondrial functionality and drastically reduce the expected increase of O_2_^•−^ that takes place as cells chronologically age. In addition, GSLs have a significant impact on glycerol catabolism, the enhancement of which has positive effects on the phosphorylating respiration state and on the reserve carbohydrate trehalose: both prerequisites for a longer chronological lifespan [32,39,69,74,75]. Additional experiments are required to elucidate the precise molecular mechanism/target underpinning GSL-mediated effects during chronological aging.

## Figures and Tables

**Table 1:** GSL extract extends CLS showing a dose–response relationship.

	Mean CLS	Max CLS	SI
NT	10.90 ± 0.52	13.99 ± 0.56	689.36 ± 60
10 µM GSL extract	10.96 ± 0.21	14.24 ± 0.33	707.98 ± 55
25 µM GSL extract	11.01 ± 0.47	14.60 ± 0.50	734.23 ± 74
50 µM GSL extract	10.89 ± 0.26	13.89 ± 0.42	771.42 ± 67
100 µM GSL extract	11.07 ± 0.39	14.76 ± 0.37	821.40 ± 31
250 µM GSL extract	11.65 ± 0.27 *	16.20 ± 0.22 *	996.97 ± 58 **
500 µM GSL extract	13.38 ± 0.50 **	18.50 ± 0.31 **	1210.46 ± 40 **
1 mM GSL extract	14.92 ± 0.21 **	21.40 ± 0.29 **	1325.25 ± 55 **
1.5 mM GSL extract	15.25 ± 0.33 **	22.15 ± 0.49 **	1340.11 ± 44 **

Data referring to the time points where chronological aging cultures of Figure 2a showed 50% (Mean CLS) and 10% (Max CLS) of survival as well as survival integral (SI) measured as in [7]. NT, untreated culture. Standard deviations are indicated (* *p* ≤ 0.05 and ** *p* ≤ 0.01).

**Table 2:** Effect on CLS of GSL extract and NAM provided together at the diauxic shift.

	Mean CLS	Max CLS	SI
NT	10.90 ± 0.52	13.99 ± 0.56	689.36 ± 60
NAM	13.06 ± 0.58 **	17.24 ± 0.49 **	979.53 ± 34 **
GSL extract	14.92 ± 0.21 **	21.40 ± 0.29 **	1325.25 ± 55 **
NAM + GSL extract	17.97 ± 0.39 **	23.28 ± 0.23 **	1510.06 ± 45 **

Data referring to the time points where chronological aging cultures reported in Figure 8b showed 50% (Mean CLS) and 10% (Max CLS) of survival as well as survival integral (SI) measured as in [31]. NT, untreated culture. Standard deviations are indicated (** *p* ≤ 0.01).

## Data Availability

Data are contained within the article.
